# Avian Influenza Virus Subtype H9N2 Affects Intestinal Microbiota, Barrier Structure Injury, and Inflammatory Intestinal Disease in the Chicken Ileum

**DOI:** 10.3390/v10050270

**Published:** 2018-05-18

**Authors:** Hongxin Li, Xiaolin Liu, Feiyang Chen, Kejing Zuo, Che Wu, Yiming Yan, Weiguo Chen, Wencheng Lin, Qingmei Xie

**Affiliations:** 1 College of Animal Science, South China Agricultural University, Guangzhou 510642, China; dongkeoffice@scau.edu.cn (H.L.); fky19842004@163.com (X.L.); cfy329@scau.edu.cn (F.C.); che.w@foxmail.com (C.W.); liaozhihong@163.com (Y.Y.); wgchen81@scau.edu.cn (W.C.); wenchenglin@scau.edu.cn (W.L.); 2 Key Laboratory of Chicken Genetics, Breeding and Reproduction, Ministry of Agriculture, Guangzhou 510642, China; 3 Key Laboratory of Animal Health Aquaculture and Environmental Control, Guangdong, Guangzhou 510642, China; 4 Guangdong Provincial Key Laboratory of Agro-Animal Genomics and Molecular Breeding, Guangzhou 510642, China; 5 Veterinary Laboratory, Guangzhou Zoo, Guangzhou 510642, China; hnlhxin@126.com; 6 South China Collaborative Innovation Center for Poultry Disease Control and Product Safety, Guangzhou 510642, China

**Keywords:** H9N2 AIV, intestinal microbiota, barrier injury, inflammatory intestinal disease, *E. coli*

## Abstract

Avian influenza virus subtype H9N2 (H9N2 AIV) has caused significant losses to the poultry industry due to the high mortality associated with secondary infections attributable to *E. coli*. This study tries to address the underlying secondary mechanisms after H9N2 AIV infection. Initially, nine day-old specific pathogen-free chickens were assigned to control (uninfected) and H9N2-infected groups, respectively. Using Illumina sequencing, histological examination, and quantitative real-time PCR, it was found that H9N2 AIV caused intestinal microbiota disorder, injury, and inflammatory damage to the intestinal mucosa. Notably, the genera *Escherichia*, especially *E. coli*, significantly increased (*p* < 0.01) at five days post-infection (dpi), while *Lactobacillus*, *Enterococcus*, and other probiotic organisms were significantly reduced (*p* < 0.01). Simultaneously, the mRNA expression of tight junction proteins (*ZO-1*, claudin 3, and occludin), TFF2, and Muc2 were significantly reduced (*p* < 0.01), indicating the destruction of the intestinal epithelial cell tight junctions and the damage of mucin layer construction. Moreover, the mRNA expression of proinflammatory cytokines IFN-γ, IL-22, IFN-α, and IL-17A in intestinal epithelial cells were significantly upregulated, resulting in the inflammatory response and intestinal injury. Our findings may provide a theoretical basis for observed gastroenteritis-like symptoms such as diarrhea and secondary *E. coli* infection following H9N2 AIV infection.

## 1. Introduction

Avian influenza virus subtype H9N2 (denoted H9N2 AIV) is found in many bird species and poultry worldwide, mostly in chickens. However, it can also infect humans and mammals and is therefore considered zoonotic [1]. Although H9N2 AIV is considered to have low pathogenicity, previous studies have shown that serious disease with high mortality and significant economic losses in broilers is associated with secondary *E. coli* infection and other pathogens [2,3,4]. Clinically, chickens infected with H9N2 AIV exhibited severe respiratory symptoms, diarrhea, and visceral inflammation including perihepatitis, pericarditis, and peritonitis, most commonly caused by *E. coli* [3,5]. After influenza infection in mammals, symptoms include cough, fever, headache, and weakness, often accompanied by gastroenteritis-like symptoms such as diarrhea [6,7]. A recent study has shown that H9N2 AIV infection alters the composition of the intestinal microbiota of chickens [8]. Using a mouse model, H1N1 influenza (PR8) infection was also shown to affect the intestinal microbial community [9]. However, the reason as to why influenza virus infection precipitates gastroenteritis-like symptoms is not yet completely understood. Moreover, there are no studies relating to how H9N2 AIV affects intestinal health or the gut microbiota.

An increasing number of reports have shown that the health of animals and humans is closely related to host intestinal microbiota. The intestinal tracts in chickens and other animals are inhabited by many diverse species of commensal microbiota that have coevolved. These organisms have derived developmental cues and their metabolic capacities strongly influence human health and biological systems [10,11,12]. Distinct components of commensal microbiota have been found within the distal digestive tract and are associated with immunological, nutritional, and pathological processes, and hence the health of the individual [13]. Therefore, when conditions in the host are unfavorable, for example, during inflammatory bowel disease, this results in an altered intestinal tract environment with consequent induction of intestinal diseases [14]. These changes have the common characteristics of reduction in obligate anaerobic bacteria and proliferation of facultatively anaerobic *Enterobacteriaceae* [15]. In most cases, the etiological relationship between the disease and intestinal microbiota has been established [14,16]. Thus, organisms and their health are habitually intertwined with the biology of the intestinal microbiota. The host’s inflammatory response and type I IFNs play critical roles in driving the changes in the microbial community structure [9,15]. Type I IFNs, including multiple IFN-α proteins and a single IFN-β protein, establish a central role in antiviral defenses in chicken and mammals [17]. However, the role of type I IFNs in bacterial defense is more ambiguous [18]. IFN-γ has a homologous effect [19]. The antiviral response to influenza may differentially sensitize hosts to secondary bacterial pneumonia.

Based on molecular biological techniques, the culture-independent approach has revealed significant diversity of microbiota in environmental samples. However, it is incomplete due to culture-based methods and conditions such as for those organisms that require a strictly anaerobic and complex environment. In recent years, second-generation sequencing methods have been widely used to accurately acquire further knowledge about the symbionts and how they work inside their host, comparing results with those of previous methods. This has enabled and completed some high-profile microbiome projects, for instance, the Human Microbiome Project [20]. Available and versatile animal models with simpler microbial communities can provide a new pattern for gut microbial symbioses [21]. In our study, we aimed to elucidate the potential associations among the intestinal microbiota, extent of barrier injury, and inflammation in the chicken ileum after H9N2 AIV infection, while also providing a theoretical basis for the prevention and control of H9N2 AIV epidemics.

## 2. Materials and Methods

### 2.1. Ethics Statement

The animal study protocol was approved by the South China Agricultural University Committee of Animal Experiments (approval ID: SYXK-2014-0136, 25 March 2014). The experiments were closely followed in accordance with the recommendations of the Guide for the Care and Use of Laboratory Animals of the National Institutes of Health. 

### 2.2. Virus Subtype and Experimental Animals

The avian influenza strain A/Chicken/Henan/SH01/2015 (SH0115) subtype H9N2 (GenBank No. KT023065) was used in all relevant experiments and isolated from chickens in poultry flocks [22], which caused high morbidity and mortality due to diarrhea and secondary bacterial infections such as *Escherichia coli*. One-day-old specific pathogen-free (SPF) chickens were purchased from WENS (Yunfu, China).

### 2.3. Animal Experiments

Thirty 1-day-old SPF chickens were randomly allocated into a control group and infection group, housed in a negative pressure isolator, and were supplied commercial food and water ad libitum. The infection group (SH01) was inoculated with 3 doses of A/Chicken/Henan/SH01/2015 at 10^6^ TCID_50_/0.1 mL through the respiratory tract at 9 days of age. The control group (mock) was inoculated with an equivalent volume of nutrient solution. Three chickens from each of the groups were euthanized and autopsied at 5 and 12 days post-infection (dpi) upon completion of the experiments. Samples from the distal ileum and the intestinal contents were collected from 1-cm ileum sections of each chicken, and then snap-frozen in liquid nitrogen. The samples from SH01 or mock were stored at −80 °C until subsequent analysis. 

### 2.4. Extraction of Metagenomic DNA

Intestinal bacterial genomic DNA was extracted with a TIANamp Stool DNA Kit (TIANGEN, Beijing, China). Total DNA TRIzol Reagent was used to extract DNA from all samples of the intestinal contents, according the manufacturer’s instructions. The DNA purity and concentration were determined using a NanoDrop spectrophotometer (Thermo, Waltham, MA, USA).

### 2.5. Illumina Sequencing

A 16S rRNA sequencing library targeting the V4 hypervariable regions was constructed according to the previous description [23,24]. The sequences of the primers were as follows: forward primer 5′-TACGTAGGGGGCTAGCGT-3′; reverse primer 5′-CCTGTTTGCTCCCCACGC-3′. Sequences have been deposited in the NCBI Sequence Read Archive under Bioproject PRJNA379944.

For HiSeq analysis, the raw data was filtered to eliminate the adapter pollution and low quality to obtain clean reads. The high-quality paired-end reads were combined to tags based on overlaps. Tags were clustered by operational taxonomic unit (OTU) at 97% sequence similarity. OTU representative sequences were taxonomically classified using Ribosomal Database Project (RDP) Classifier v.2.2 (East Lansing, MI, USA). Finally, alpha diversity, beta diversity, and the different species that were screened were analyzed based on OTU and taxonomic ranks. 

### 2.6. Histological Examination of Intestinal Segments and Villus Conditions

The distal ileum was sectioned for evaluation of the ileal epithelium lesions. Each piece was fixed in 10% neutral-buffered formalin for 24 h and embedded in paraffin. Sections of 4-mm thickness were stained with hematoxylin and eosin stain (H&E stain). Intestinal tissue slice was observed using the microscope. Ten complete structures of the villi and crypt depth were measured using Image-Pro Plus 6.0 (Media Cybernetics, Silver Spring, MD, USA), and the villus length and crypt depth (V/C) ratios were calculated.

### 2.7. Extraction of Total RNA

Total RNA was extracted from the ileal epithelium samples with TRIzol (Invitrogen, Carlsbad, CA, USA) following the manufacturer’s instructions, and cDNA was synthesized according to protocols as previously described [25].

### 2.8. Quantitative Real-Time PCR

Quantitative real-time PCR was performed for the *Escherichia* bacterial count. The total volume of DNA extracted was derived from 1-cm sections of the ileal mucosa and contents, and then the DNA was adjusted to the same concentrations. DNA standards were prepared from *E. coli* strains carrying plasmids with *E. coli* fragment inserts, which was isolated from a poultry farm (GenBank No. MG602206). The abundance of the gene was evaluated by multiplying the number of copies per well by the total volume of DNA per well (1.0 μL). The total reaction volume of 20 μL contained 1.0 μL DNA, 10.0 μL SYBR Green qPCR Mix (Roche Diagnostics, Shanghai, China), and 0.5 μL of each primer (forward and reverse, see Table 1 for sequences). 

The relative expression levels of the target genes (see Table 1 for sequences) expression was evaluated by qPCR. For analysis, target gene expression of each sample was normalized to the reference gene, glyceraldehyde-3-phosphate dehydrogenase (GAPDH), following a previously described protocol [26]. The 2^−^^△△Ct^ method was used to analyze the results of the qPCR.

### 2.9. Determination of Butyrate

The butyrate extraction procedure and determination were performed was according to the previous description [27] in Guangdong Longsee-med Bio Medicai., Ltd. (Guangzhou, China). For the determination of butyrate, we used 500 mg of the frozen fecal samples. 

### 2.10. Statistics

Data analysis was conducted using GraphPad Prism (version 5.0, La Jolla, CA, USA) and are expressed as the mean ± SE. Unless otherwise noted, the differences between treatment groups were analyzed using a Student’s two-tailed *t*-test and one-way analysis of variance (ANOVA). Differences were considered to be statistically significant when *p* < 0.05.

## 3. Results

### 3.1. H9N2 AIV Infection Causes Intestinal Structure Injury

**Figure 1:**
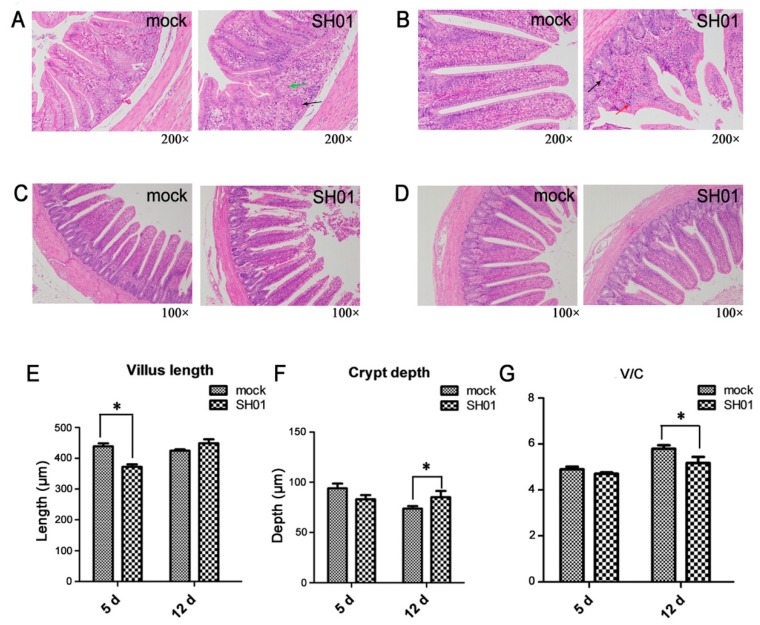
Histopathological changes in the ileal mucosa. (**A**,**B**) Histological features in the mock and SH01 groups are shown with hematoxylin and eosin staining at 5 dpi (d) and 12 dpi. Degeneration and necrosis of crypt cells are indicated with the black arrow. Lymphocytic infiltration is indicated with the green arrow. Degeneration of the mucosal epithelial cells and pyknosis are indicated with the red arrow. (**C**,**D**) Images at lower magnification (100×) for histological observation and statistics are provided. (**E**,**F**) Measurements of villus length (**E**) and crypt depth (**F**) by Image-Pro Plus 6.0 (mock = 6, SH01 = 6). (**G**) Spatial distribution of villus-length/crypt-depth of mock and SH01 groups. * *p* < 0.05.

To test whether intestinal injury was a feature of chickens infected with the H9N2 AIV strain (A/Chicken/Henan/SH01/2015), we firstly detected live virus from cloacal swabbing. The peak of virus shedding was observed at 5 dpi. Ileac histopathology analysis showed that degeneration and necrosis of crypt cells were found in the infection group SH01 (Figure 1A,B). Local lymphocytic infiltration was observed in SH01 at 5 dpi (Figure 1A). There was degeneration of the mucosal epithelial cells and pyknosis in SH01 at 12 dpi (Figure 1B). Histological observation and statistics were provided by images at lower magnification (Figure 1C,D). The villus length of SH01 was significantly reduced (*p* < 0.05, Figure 1E) at 5 dpi, and the crypt depth of SH01 was significantly increased (*p* < 0.05, Figure 1F) at 12 dpi. The mucosal regular villus-length/crypt-depth (V/C) was significantly reduced (*p* < 0.05, Figure 1G) at 12 dpi, indicating that the ileal villi and crypts suffered varying degrees of injury after infection.

### 3.2. Intestinal Bacterial Microbiota Composition Differs between H9N2-Infected and Control Groups

**Figure 2:**
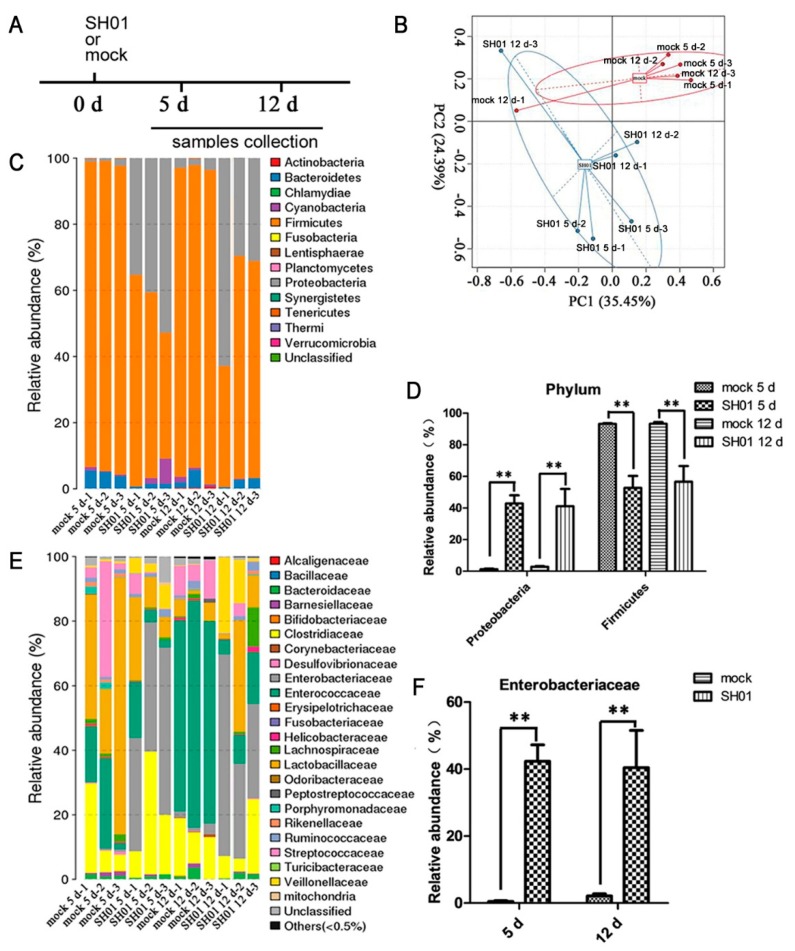
H9N2 AIV infection alters the intestinal microbiota composition. Analysis of the ileal microbiota in mock and SH01 groups by HiSeq sequencing. (**A**) Experimental model: the samples were collected from chickens at 5 dpi and 12 dpi. (**B**) Principal component (PC) analysis based on operational taxonomic unit (OTU) abundance using software R (v3.1.1). A dot represents each sample, the red represents the mock group, and the blue represents the SH01 group. (**C**,**E**) The ileal microbiota from mock and SH01 groups at 5 dpi and 12 dpi before infection (*n* = 6 for both mock and SH01 groups) was analyzed by sequencing using the Illumina HiSeq system. The relative abundance of the bacterial phylum (**C**) and family (**E**) is displayed; the cutoff abundance level was set at 0.05%. (**D**) Spatial distribution of microbial composition at the major phylum level. (**F**) Spatial distribution of microbial composition at the family level of Enterobacteriaceae. ** *p* < 0.01.

SPF chickens were monitored daily for 12 dpi. We assessed the microbiota composition in the ileal contents (containing both mucosal and lumen microbes) of SH01 and mock groups at 5 dpi and 12 dpi (Figure 2A), as respiratory symptoms mostly appeared after 4 days post-infection. Principal component analysis (PCA) showed that the microbial communities in SH01- and mock-infected chickens could be separated using the operational taxonomic unit (OTU) composition dataset (Figure 2B), indicating that these groups had significantly different bacterial compositions. Firmicutes and Proteobacteria comprise two major bacterial phyla in all samples (Figure 2C). The proportion of Firmicutes after 5 dpi significantly decreased (*p* < 0.01) in the SH01 group, while the quantity of Proteobacteria was significantly upregulated (*p* < 0.01, Figure 2D), suggesting that some beneficial bacteria may be significantly inhibited by the growth of some pathogens. Specifically, members of the Enterobacteriaceae in the Proteobacteria phylum were significantly augmented (Figure 2E,F) after H9N2 AIV infection, which has been associated with markers of mucosal inflammatory and immune disruption, as observed in previous reports [28,29,30]. This indicated that the phenomenon is associated with mucosal inflammation in the ileum after H9N2 AIV infection.

### 3.3. Abundance of E. coli Increased Sharply in the Ileum after H9N2 AIV Infection

**Figure 3:**
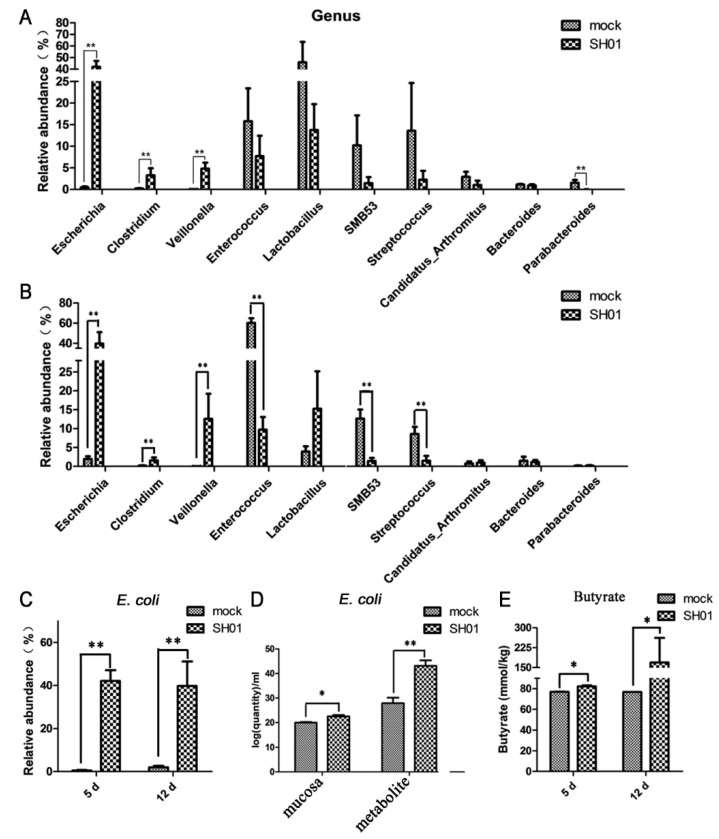
H9N2 AIV infection mainly affects the relative abundance of 10 variable taxa at the genus level and relative abundance of *E. coli* at the species level. (**A**) Spatial distribution of microbial composition at the major genus level at 5 dpi. (**B**) Spatial distribution of microbial composition at the major genus level at 12 dpi. (**C**) Spatial distribution of microbial composition at the genus level of *E. coli*. (**D**) Quantity of *E. coli* in ileal contents of mock and SH01 groups by real-time RT-PCR. (**E**) Quantity of butyrate in ileal contents of mock and SH01 groups. ** *p* < 0.01, * *p* < 0.05.

Our findings suggest that endogenous Enterobacteriaceae were abnormally increased after H9N2 AIV infection, which corroborates similar findings for influenza virus infection [9]. We were interested in detecting the most active bacteria, excluding any exogenous pathogenic bacterial infections. Analysis of the genus of 16S rDNA libraries indicated that several genera significantly contributed to the dissimilarities in the community compositions among different individuals. *Escherichia*, *Clostridium*, and *Veillonella* increased significantly (*p* < 0.01, Figure 3A,B) in the ileum after H9N2 AIV infection. In contrast, the amount of *Enterococcus*, *Lactobacillus*, *Streptococcus*, *SMB53*, *Candidatus* Arthromitus, *Bacteroides*, and *Parabacteroides* decreased, especially lactic acid-producing bacteria (i.e., *Enterococcus* and *Streptococcus*) and *SMB53* (Figure 3A,B). Notably, we demonstrated that *Escherichia coli* significantly increased (*p* < 0.01, Figure 3C), and the median relative abundances of *Escherichia coli* were 42.07% at 5 dpi and 39.78% at 12 dpi, respectively. The quantity of *Escherichia coli* was detected by qPCR to further verify the result of 16S rDNA sequencing metagenomics, and results showed that *Escherichia coli* in ileal mucosa and ileal metabolites significantly increased (*p* < 0.01, Figure 3D). These data indicate that H9N2 AIV infection of the respiratory tract causes the explosion of *Escherichia coli*. In addition, the ileal contents were collected from SH01 and mock, and the butyrate levels were quantified by liquid chromatography and mass spectrometry. The ileal contents from SH01 contained more butyrate than the samples from mock chickens (*p* < 0.05, Figure 3E), suggesting that butyrate might play a role in colonization by *Escherichia coli* after H9N2 AIV infection. 

### 3.4. H9N2 AIV Infection Damages Ileal Mucous Layer Construction and Tight Junctions

**Figure 4:**
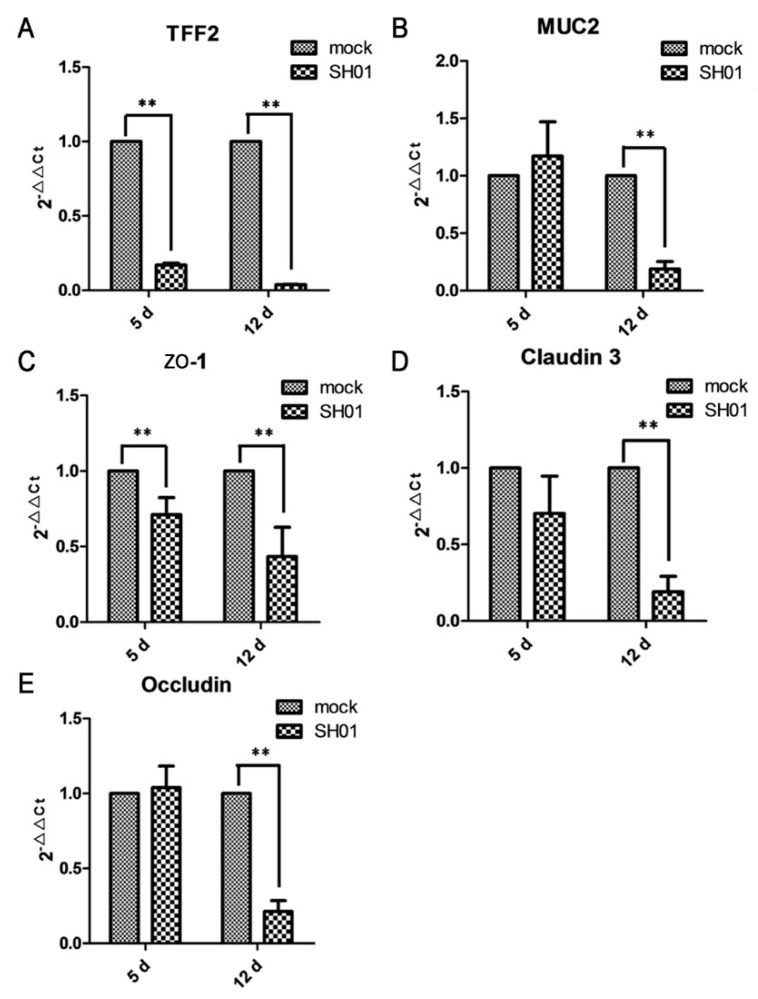
H9N2 AIV infection affects TFF2, MUC2, *ZO-1*, claudin 3, and occludin gene expression in the ileal epithelial cells of mock and SH01 groups, as found by real-time RT-PCR. (**A**) TFF2 gene expression at 5 dpi and 12 dpi. (**B**) MUC2 gene expression at 5 dpi and 12 dpi. (**C**) *ZO-1* gene expression at 5 dpi and 12 dpi. (**D**) Claudin 3 gene expression at 5 dpi and 12 dpi. (**E**) Occludin gene expression at 5 dpi and 12 dpi. ** *p* < 0.01.

Since mucins and trefoil peptides are key components of mucus, we examined the effect of H9N2 AIV infection on the expression of these mucous layer proteins. TFF2, a secreted protein of the gastrointestinal mucosa, was significantly downregulated (*p* < 0.01) at 5 dpi and 12 dpi (Figure 4A). MUC2, a protective and antimicrobial mucoprotein, was also significantly downregulated (*p* < 0.01) at 12 dpi (Figure 4B). These results indicated that H9N2 AIV infection of the respiratory tract impacts upon the ileal mucous layer construction. The mRNA expression of *ZO-1*, a cytoplasmic protein, was significantly decreased (*p* < 0.01, Figure 4C) at both 5 dpi and 12 dpi, while expression of claudin-3, a cell membrane protein, and occludin, which is related to the cell membrane permeability, were both significantly decreased (*p* < 0.05, Figure 4D,E) at 12 dpi. The results indicate that mucosal epithelial cell tight junctions are injured after H9N2 AIV infection.

### 3.5. H9N2 AIV Infection Promotes mRNA Expression of Proinflammatory Cytokines IFN-γ, IL-22, IFN-α, and IL-17A

**Figure 5:**
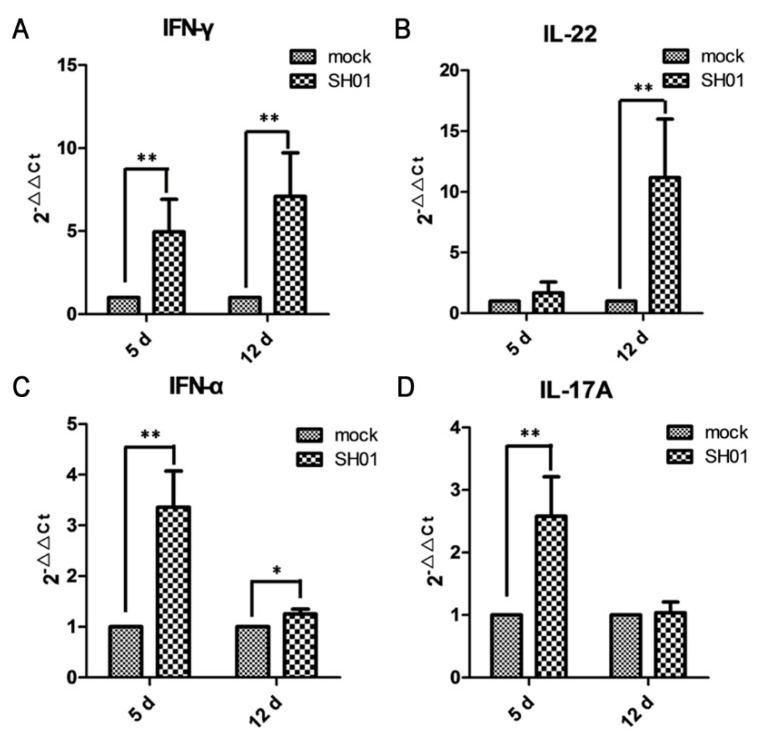
H9N2 AIV infection promotes mRNA expression of proinflammatory cytokines IFN-γ, IL-22, IFN-α, and IL-17A, as found by real-time RT-PCR. (**A**) IFN-γ expression at 5 dpi and 12 dpi; (**B**) IL-22 expression at 5 dpi and 12 dpi; (**C**) IFN-α expression at 5 dpi and 12 dpi; (**D**) IL-17A expression at 5 dpi and 12 dpi. ** *p* < 0.01, * *p* < 0.05.

Given that inflammatory tissue is usually associated with variations in the level of expressed cytokines, we aimed to test whether H9N2 AIV infection promoted intestinal epithelial cell proinflammatory expression. IFN-γ, an antiviral and immunomodulatory cytokine in chickens, was significantly upregulated at 5 dpi and 12 dpi compared with mock-infected chickens (*p* < 0.01, Figure 5A). IL-22, a pivotal mediator in regulating tissue responses during inflammation in chickens [31], was significantly upregulated at 12 dpi (*p* < 0.01, Figure 5B). Furthermore, IFN-α was significantly upregulated (Figure 5C) at 5 dpi (*p* < 0.01) and 12 dpi (*p* < 0.05) compared with mock, which could be related to alterations observed in the intestinal microbiota profile in mice [9]. IL-17A is related to intestinal injury [19,32] and was significantly upregulated after 12 dpi (Figure 5D, *p* < 0.01) compared with mock. These data imply that an antiviral response to influenza might differentially sensitize hosts to *Escherichia coli* organisms.

## 4. Discussion

The intestine is the natural habitat for a large and dynamic microbial community. The intestinal microbiota is helpful to health and profoundly influences the normal structural and functional development of the mucosal immune system. Therefore, it is very important to understand the quantity and quality of intestinal microbiota and how the composition can change with H9N2 AIV infection. Moreover, a recent report has stated that the composition of the fecal microbiota of chickens is disordered during H9N2 AIV infection [8], but the composition of the intestinal microbiota and the mechanism by which the influenza virus can affect the intestinal microbiota are unclear. In this study, we observed chicken intestinal microbiota consisting of three major bacterial phyla: the Firmicutes, the Proteobacteria, and the Bacteroidetes, which confirmed previous observations [33]. The fact that the H9N2 AIV infection increased the relative abundance of the Enterobacteriaceae, particularly *E. coli*, is similar to the change of intestinal microbiota observed in Crohn’s disease [9,30]. In line with intestinal inflammation in mammals, in which facultative anaerobic Enterobacteriaceae tend to thrive, the abundance of obligate anaerobes was reduced [28,29,34]. This research is in accordance with the findings that obligate anaerobic lactic acid-producing bacteria (i.e., *Enterococcus*, *Lactobacillus*, and *Streptococcus*) were significantly reduced. 

Type I IFNs are mainly considered to be antiviral and immunomodulatory cytokines in chickens [17]. It has been reported that avian type I IFNs are effective antiviral agents and can inhibit Marek’s disease virus (MDV), infectious bronchitis virus (IBV) [35,36], and H9N2 AIV infection [37]. The studies showed that IFN-α and Proteobacteria were both significantly increased, suggesting that IFN-α plays a role against H9N2 AIV and has no positive effect on the intestinal microbiota. Moreover, type I IFNs had been considered to promote the blooming of indigenous Proteobacteria in mice [9]. Further research in the chicken animal model is needed.

IL-17A was reported to participate in the induction of inflammation in chickens during intestinal infection [32,38]. It was significantly increased in ileal epithelial cells, suggesting that inflammation had occurred. Simultaneously, there was severe mucosal injury, identified by the analysis of mucosal morphology and histochemistry after 5 dpi. In addition, IFN-γ and IL-22 were also significantly increased after H9N2 AIV infection. Similar to its mammalian counterparts, chicken IFN-γ triggers the release of the production of nitric oxide and forming of nitrate in the lumen [39,40]. *E. coli* could rapidly multiply through nitrate respiration [41]. Chicken IL-22 can modulate tissue responses during inflammation [31]. In mammals, IL-22 triggers the release of antimicrobial lipocalin-2, which blocked the growth of commensal Enterobacteriaceae, but not *E. coli* [40]. Based on these results and previous reports, we infer that anaerobes, such as Firmicutes, comprise the significant majority of healthy microbiota in the ileum. However, H9N2 AIV infection promotes the growth of indigenous Proteobacteria, especially *E. coli*, to the apparent detriment of restricted anaerobic commensals, leading to significant intestinal inflammatory disorders. While the chicken cytokines remain poorly understood and their biological activities may be numerous, nevertheless, we can leave it as a possibility which requires further verification.

Mucins are protective, antimicrobial substances, which are secreted by epithelial cells. MUC2 is the primary gel-forming mucin in the mammalian gut [42]. Endogenous trefoil (TFF) peptides are a class of secreted proteins of the intestinal mucosa, which can inhibit apoptosis [43] and reduce antigen access to the healing epithelium [44]. The pathogenic bacterium (including *Eimeria* and *Clostridium perfringens*) could suppress the transcriptional level of MUC2 and TFF2 in chickens [45,46]. Our findings showed that MUC2 and TFF2 were significantly decreased, suggesting the injury of the mucus layer and the invasion of *E. coli*. Moreover, TFF2 can inhibit inducible nitric oxide synthase (iNOS) in monocytes and inflammatory compartments and regulate monocyte NO-mediated inflammation in colitis [47]. Therefore, we proposed that the formation of nitrate was increased.

The intestinal epithelium provides a selective permeable barrier to limit noxious molecules and absorb nutrients and water. Intercellular tight junction (TJ) structures conduct precisely this selective function. TJ structures are multiple protein complexes, comprising occludin [48], claudins [49], and three ZO proteins [50,51,52], located at the apical ends of the intestinal epithelial lateral membranes of cells [53]. Previous studies showed that the intestinal TJ has a vital role in the pathogenesis of intestinal pathogens [45,54]. Disruption of the TJ barrier promoted paracellular permeability and then absorbed luminal proinflammatory molecules by permeation, which caused tissue damage and inflammation. In this study, we observed significant decreases in *ZO-1*, claudin-3, and occludin, which suggested that the ileal TJ structures were destroyed after 5 dpi. Pathogens and their associated virulence factors increased the opportunity to enter the intestinal structure and the blood circulation system through paracellular permeability. Consequently, these can enhance mucosal inflammation or may be a critical factor for induction of inflammation after H9N2 AIV infection, resulting in perihepatitis and pericarditis. 

Butyrate, a short-chain fatty acid, accounts for about 70% of the energy source for colonic enterocytes [55,56]. Some studies of chickens indicated that butyrate promotes the growth of chickens and suppressed colonization by *Salmonella* [57], but it may be detrimental to the host under certain conditions. Butyrate could facilitate the host’s expression of globotriaosylceramide, a receptor for Stx [58]. Our findings showed that ileal butyrate production was significantly increased after H9N2 AIV infection, suggesting that the function of butyrate may play multiple roles in the chicken intestine. On the other hand, butyrate is mainly produced in the cecum in chickens [59], and the distal ileum was used for evaluation in the study. Thus, it may be a self-regulating capacity of the host against H9N2 AIV infection.

Collectively, H9N2 AIV infection enhanced the expression of proinflammatory cytokines, such as IFN-α, IL-17A, IFN-γ, and IL-22, and promoted the proliferation and translocation of Proteobacteria, especially *E. coli*, which might be induced by the injury of mucous layers and tight junctions. This research provides new insight for the research of the molecular mechanism of H9N2 AIV and a theoretical basis for the control of this disease.

## Figures and Tables

**Table 1:** Sequences of RNA oligonucleotides.

Name	Sense Strand/Sense Primer (5′–3′)	Antisense Strand/Antisense Primer (5′–3′)
TFF2	CCCTGCTGATCCTCGTAT	GCTGTTATTTCCCAGTTGA
MUC2	AATGCTGAGTTCTTGCCTAA	GTTGCAGTTCATATCCTGGT
*ZO-1*	GCCTGAATCAAACCCAGCAA	TATGCGGCGGTAAGGATGAT
Claudin-3	GAAGGGCTGTGGATGAACTG	GAGACGATGGTGATCTTGGC
Occludin	GATGGACAGCATCAACGACC	CATGCGCTTGATGTGGAAGA
IFN-γ	ATCATACTGAGCCAGATTGTTTCG	TCTTTCACCTTCTTCACGCCAT
IL-22	CAGGAATCGCACCTACACCT	TCATGTAGCAGCGGTTGTTC
IFN-α	CCAGCACCTCGAGCAAT	GGCGCTGTAATCGTTGTCT
IL-17A	CCATTCCAGGTGCGTGAACT	TTTCTTCTCCAGGCGGTACG
GAPDH	AGGCTGAGAACGGGAAACTTG	CACCTGCATCTGCCCATTTG
*E. coli*	GTTAATACCTTTGCTCATTGA	ACCAGGGTATCTTAATCCTGTT
